# An Improved Speed Sensing Method for Drive Control

**DOI:** 10.3390/s25020515

**Published:** 2025-01-17

**Authors:** Manuel R. Arahal, Manuel G. Satué, Juana M. Martínez-Heredia, Francisco Colodro

**Affiliations:** 1 Departamento de Ingeniería de Sistemas y Automática, Universidad de Sevilla, 41092 Seville, Spain; 2 Departamento de Ingeniería Electrónica, Universidad de Sevilla, 41092 Seville, Spain

**Keywords:** induction motor, multi-phase IM, predictive control, speed sensing

## Abstract

Variable-speed electrical drive control typically relies upon a two-loop scheme, one for torque/speed and another for stator current control. In modern drive control methods, the actual mechanical speed is needed for both loops. In practical applications, the speed is often acquired by incremental rotary encoders. The most used method derives speed from an encoder pulse count during a fixed amount of time. It is known that this sensing method produces time delay in the speed feedback loop as well as fluctuations in the speed measurements. Time lags produce phase loss that has potentially negative effects on the overall drive performance. Nevertheless, the pulse counting method is favored in most cases due to its simplicity and existing support for its use in digital signal processors. In this paper, a new speed sensing method is proposed to reduce time lag without incurring increased fluctuations. The proposal uses a novel transient detector to determine the actual operational regime of the drive: transient or stationary. Transient detection is not based on measured speeds but works directly with the train of incoming encoder pulses. The method is designed to work well with established digital signal processor routines. The proposal is assessed through experimentation on a real five-phase induction motor.

## 1. Introduction

Induction motors (IMs) are used in many applications in industry, in transportation, and at home [1]. Speed regulation is achieved by using an electronic power converter that supplies a sinusoidal-like voltage. Indirect Field-Oriented Control (IFOC) is a now-mature technology for drive control [2]. IFOC tuning has been investigated using stability methods [3]. The rotor time constant has been found to play an important role [4]. However, these analyses use a simplified dynamic model [5], not considering speed sensing.

Most practical applications rely on simple speed sensing derived from incremental encoders. In laboratory and high-end products, Digital Signal Processors (DSPs) are used. These utilize special routines for encoder treatment. Although more sophisticated methods exist [6,7,8], there is resistance to their adoption. This resistance is higher for methods requiring abandoning ready-made DSP modules based on counting by interruptions.

Some papers have considered the performance of basic speed sensing using quadrature encoders [9]. A survey of new techniques is presented in [10,11]. Some of the improvements rely on increasing the sampling frequency [12,13], opening the path for noise shaping [14]. Other works have concentrated on new sensing models for improved control [15].

The relevance for such research is not restricted to the strict IFOC method. The IFOC structure is also present in Finite-State Model-Predictive Control (FSMPC) [16]. FSMPC uses the IFOC idea but replaces the inner PI and PWM blocks with a predictive controller [17]. This has enabled the easy use of IFOC-like structures in applications such as multi-phase drives [18]. This is relevant as multi-phase systems are being proposed to replace three-phase ones in applications needing fault tolerance and better energy distribution [19].

The FSMPC method is becoming popular for its flexibility. For instance, modulation variants that are impossible with PWM can be used with FSMPC [20,21,22], the mechanical load characteristics can be considered with ease, and fault-tolerance capabilities are better utilized [23]. State sensing methods such as Kalman filtering and observers can also be incorporated with ease in MPC approaches [24,25], expanding their potential with respect to the PI case. Finally, the high computational demand of FSMPC has been drastically reduced by the introduction of several fast computation algorithms [26,27,28,29]. This has made FSMPC a strong contender for traditional methods based on the two-loop scheme, even replacing both loops [30].

Recent proposals for speed sensing include methods for the efficient realization of low-cost platforms avoiding arithmetic division operation [7]. In the present context, the use of DSP is necessary for FSMPC, so the division operation places no challenge. In [31], a trigonometric Kalman filter is used as a signal fusion technique to provide rapid angle estimations for a multi-head rotational encoder. The method, however, deviates a lot from interruption-based pulse counting, which hinders its adoption. Regarding the interaction of speed sensing with speed control, the work of [32] proposes cascade proportional integral retarded control where the inner loop uses an integral retarded law and the outer loop a proportional law for angular control.

In this paper, a new speed sensing method for DSP is proposed. The proposal relies on the interruption-based counting structure of DSP. In this way, its adoption is made easier. In the context of the above-presented state of the art, the contributions of this paper can be summarized as follows.

1. The effect of counting-based speed sensing is modelled, including the time lag and variance of measurements.
2. A new speed sensing is proposed using the popular interruption-based DSP module. The proposal reduces the time lag without incurring increased variance in the measurements.
3. Controller tuning with improved speed measurements is experimentally investigated.

The next section provides background on speed sensing using encoders. Then, in Section 3, the proposal is presented. In Section 4, the proposal is tested experimentally.

## 2. Speed Sensing via Incremental Encoders

Incremental encoders produce electrical pulses as the mechanical axis rotates. The pulses are captured by DSP hardware. This information is then handled by an interruption routine that is run every $T_{\omega }$ seconds. The number of pulses that have arrived since the last interruption is denoted as Δx. The standard speed sensing is then computed as

(1) $$\hat{\omega }=k_{\omega }\frac{\Delta x}{T_{\omega }}.$$

In the above equation, the factor $k_{\omega }$ is a parameter depending on *Q*, the number of pulses per revolution that the encoder can issue. If speed is measured in rad/s, then $k_{\omega }$ is computed as $k_{\omega }=2\pi /Q$. High-end encoders have *Q* in the range of many thousands of pulses per revolution.

The standard speed sensing provided by (1) is by far the most commonly found method in drives using an incremental rotary encoder. An analysis is in order to set the bar for possible improvements. First of all, please note that the value provided by (1) for the constant speed ω can change from one interruption period to another. The number of pulses for a given $T_{\omega }$ is $\Delta x^{t}=\frac{\omega T_{\omega }}{k_{\omega }}$, which can be a non-integer. In some periods, one obtains a value over $\Delta x^{t}$, and in other periods, a value below $\Delta x^{t}$ is obtained. This produces fluctuations in the sensed speed $\hat{\omega }$. The deviation $\delta =(\omega -\hat{\omega })$ can take only two values: $\delta_{1}=k_{\omega }/T_{\omega }\left(\left\lfloor \Delta x^{t}\right\rfloor \right)$ and $\delta_{2}=k_{\omega }/T_{\omega }\left(\left\lfloor \Delta x^{t}\right\rfloor +1\right)$. The first value for δ is found with the probability $r=1-\Delta x^{t}/\left\lfloor \Delta x^{t}\right\rfloor$ and the second value with the probability 1−r. This allows the computation of $\sigma_{\omega }^{2}$ as

(2) $$\sigma_{\omega }^{2}={\left(\frac{k_{\omega }}{T_{\omega }}\left(r\delta_{1}+\left(1-r\right)\delta_{2}\right)\right)}^{2}$$

**Figure 1:**
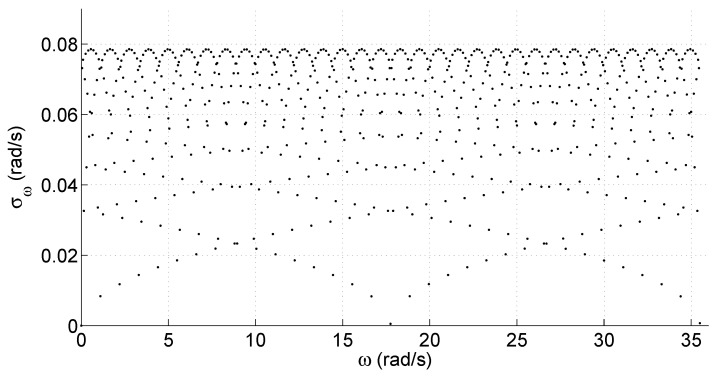
Standard deviation for standard speed sensing provided by (1) as a function of the real speed ω.

In this way, it is possible to compute the standard deviation for the speed measurements $\sigma_{\omega }$ as a function of ω. This was conducted for several ω values, using (2) to produce the graph of Figure 1. For the computations, a value of $T_{\omega }=10$ (ms) was used for illustrative purposes. The incremental encoder used had 4000 ppr. The actual values did change the distribution quantitatively but not qualitatively.

The distribution of $\sigma_{\omega }$ shown in Figure 1 is periodic. The graph in the figure shows just the first cycle. A somewhat chaotic distribution is found that stems from the integer rounding of the actual measurements.

**Figure 2:**
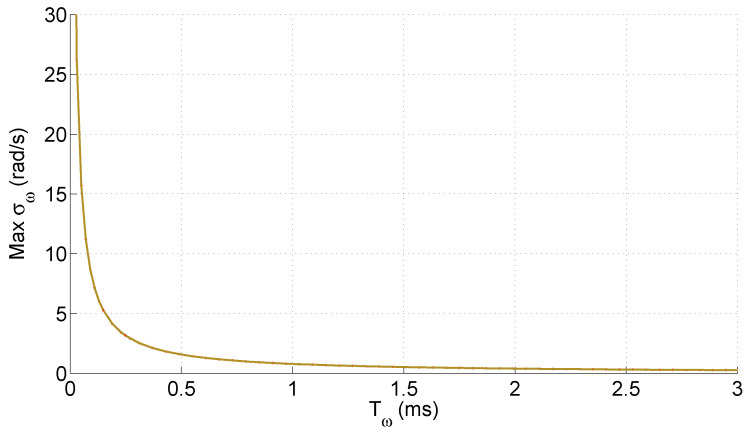
The maximum value for the standard deviation $\sigma_{\omega }$ as a function of the interruption period $T_{\omega }$.

In most papers, a medium value, found by simulation, is reported. For variable-speed drives, all speeds must be considered. As a result, one must be prepared to deal with the maximum $\sigma_{\omega }$. This maximum value does not depend on ω, as has been correctly reported in previous works [9]. However, its dependency on $T_{\omega }$ is known to be a hyperbola, as shown in Figure 2. This means that the use of a higher $T_{\omega }$ is a strategy with diminishing returns. The hyperbola of Figure 2 was obtained using the maximum value found with (2) for each $T_{\omega }$.

This problem is exacerbated for low values of ω. This is because in one interruption cycle, Δx can be zero even if the actual speed is not. To overcome this problem, one could resort to increasing $T_{\omega }$, but this works badly for higher speeds.

The above exposed problems have led to temporal filtering as a simple solution. Then, the values provided by (1) are subject to low-pass filtering. The most commonly found method for this is provided by the first-order digital filter that takes the form

(3) $${\hat{\omega }}^{f}\left(k\right)=a\cdot {\hat{\omega }}^{f}\left(k-1\right)+\left(1-a\right)\cdot \hat{\omega }\left(k\right),$$

where ${\hat{\omega }}^{f}$ is the filtered speed and *a* is a parameter with 0<a<1. In this way, the oscillations found in the measurements are smoothed as the low-pass filter reduces the amplitude of high-frequency signals. This type of filter is easily programmed into the DSP even inside the interruption that handles the encoder counting. The value *a* is found as a trade-off between fast sensing (for low values of *a*) and reduced oscillations (for higher values of *a*). The choice must take into account the characteristics of the encoder, namely the number *Q* of pulses per revolution and the interruption period $T_{\omega }$.

Please note that filters of higher orders can be used. However, in practical applications, simple solutions are more often than not adopted. Nevertheless, the proposal can work with any other type of filter as well.

Other approaches for speed sensing using an incremental encoder are based on the times of arrival of pulses. Then, instead of fixing the interruption time, it is the time between successive pulses that is measured. In this way, the speed can be found as

(4) $$\hat{\omega }=k_{\omega }\frac{1}{\Delta T}.$$

This method works well for low speeds. At high speeds, ΔT becomes to small to be accurately measured, incurring an error.

Of course, mixed methods do exist in which both sensed speeds are combined to produce an improved measurement that works well for stationary regimes both at low and at high speeds. The challenge that remains is producing speed measurements for transient regimes.

## 3. Proposed Speed Sensing Method

To enhance the speed sensing provided by interruption-based counting methods, one should have to consider several factors.

Operating regimes with a constant ω are termed stationary regimes (SRs). In SRs, the main factor is the variance of the sensed speed. For transient regimes (TRs), the main factor is the time lag associated with the sensing process. High values of $T_{\omega }$ and low-pass filtering both have a positive effect on SRs but cause latency that is pernicious for TRs. Conversely, low values of $T_{\omega }$ and no filtering reduce latency in TRs at the cost of increased variance for SRs.

The proposal uses a novel transient detector to determine the actual operational regime of the drive: transient or stationary. Transient detection is not based on measured speeds as in previous proposals but works directly with the train of incoming encoder pulses. In this way, transient detection is made faster, since it does not involve the lag intrinsic to speed sensing.

A transient detector uses the values y(i)=Δx(i) where the index *i* refers to the interruption. Do not confuse it with the index *k* corresponding to the sample time of the control routine. A min–max operator is applied to a set, *Y*, of values, y(j), corresponding to the interruption numbers j=i, j=i−1, *…*j=i−L+1, where *L* is a parameter to be established later. As a result, the transient indicator γ is found as

(5) γ(i)=maxy(j)−miny(j),∀y∈Y

During a stationary regime, the values found by (5) verify γ≤1. This is a consequence of the analysis performed for the interruption-based counting process. On the other hand, for transient regimes, one finds the values γ>1.

Regarding DSP resources, the computation indicated by (5) requires just storing the last *L* values provided by the interruption-based pulse counter. This requires an affordable quantity of memory as *L* is not large, as will be shown later.

The proposed method uses a transient indicator to determine how to measure speed. For transient regimes, fast sensing is enabled to reduce the time lag in the speed sensing. For non-transient regimes, slower sensing is enabled to attain reduced variance.

To determine the parameters for the method, one can resort to Equation (2) and select a value, $T_{\omega }^{base}$, that produces variance that is small enough for the application according to the parameter *Q* of the encoder being used. For instance, in the scenario considered for Figure 2, a value of $T_{\omega }^{base}=3$ ms could be appropriate. After this, the interruption for the pulse counting must be set to $T_{\omega }=T_{\omega }^{base}/L$. The time window for the transient detector should use a handful of interruption periods, so 2<L<10 is a rule of thumb.

In some applications (such as compressors), changes in the reference speed are never steep but are provided in the form of ramps. The slope of the ramp can be used to determine the value of *L*. Steeper slopes can be managed with smaller values for *L*. Conversely, for smooth transients, one might require higher values of *L*.

After these parameters are chosen, the method can be realized by the DSP pulse counting routine using the following steps.

1. Obtain the actual value y(i).
2. Roll the stored *y* values in *Y* to include y(i), discarding y(i−L).
3. Apply the min–max operator to the updated set *Y*, computing γ(i).
4. If γ(i)≤1, use $\hat{\omega }=k_{\omega }\sum y/T_{\omega }^{base}$ or else use $\hat{\omega }=k_{\omega }y\left(i\right)/T_{\omega }$.

The computational load for these extra steps (compared to the standard method) is negligible for a DSP-based application. Also, as these computations take place in the interruption-based counting routine, they do not affect the speed control routine that runs at a much higher sampling frequency.

## 4. Experimental Assessment

To test the proposal, an experimental laboratory test bed was used. The setup consisted mainly of a five-phase induction machine that was controlled using a DSP. The control method is detailed below and the experimental setup is detailed in the following subsection.

### 4.1. Drive Control

The ultimate goal of the speed sensing method is to provide a feedback signal for speed control. The control loop relies on a proportional integral (PI) controller that handles speed tracking. This control loop is carried out by the DSP in discrete time. An external speed reference signal has to be supplied; this is represented as $\omega^{*}\left(k\right)$. Then, the speed tracking error is found as $e\left(k\right)=\omega^{*}\left(k\right)-\hat{\omega }\left(k\right)$. The speed tracking error is used to derive the reference for the quadrature stator current. By denoting the quadrature stator current as $i_{q}$, the reference value is found as

(6) $$\begin{array}{c} i_{q}^{*}\left(k\right)=k_{p}\cdot e\left(k\right)+k_{i}\sum_{j=0}^{k}\;e\left(j\right) \end{array}$$

where $k_{p}$ and $k_{i}$ are the PI gains and $i_{q}^{*}$ is the reference for the quadrature stator current, i.e., the current in the axis *q* (torque-producing). The PI gains can be set using any PI tuning method. The reader can consult [33] for a review. The values used in the experiments are provided in Section 4.4. Now, for the reference of the current in the *d* axis, the nominal flux $\Phi_{n}$ is normally used as

(7) $$\begin{array}{c} i_{d}^{*}\left(k\right)=\Phi_{n}/L_{m} \end{array}$$

where $L_{m}$ is the mutual inductance.

With Equations (6) and (7), the reference for the stator d−q current is established. Now, the voltages are set by the VSI so that the actual current follows the references. This is performed by an inner feedback loop. Various methods have been proposed for this, such as IFOC [2] and Finite-Set Model-Predictive Control [17], to name two of the most popular ones.

Is important to notice that having an accurate measurement of speed is essential, as measurement ripple affects both the external and internal control loops. Also, the speed of response to changes in the actual speed affects the speed loop.

### 4.2. Experimental Setup

**Figure 3:**
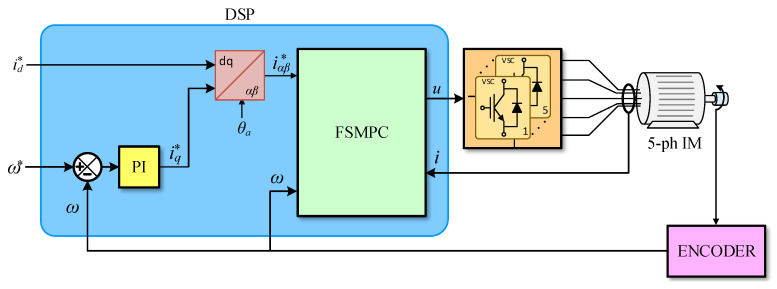
A diagram of the speed control system used for the assessment of the speed sensing proposal.

**Figure 4:**
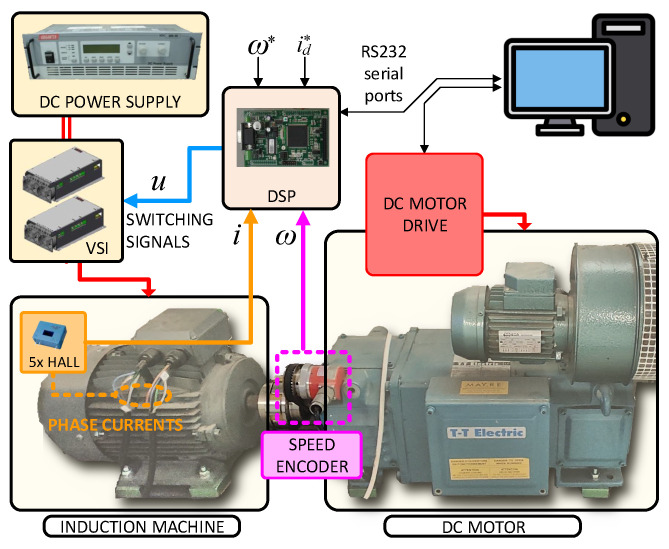
Elements of the experimental five-phase drive.

The experimental setup used a five-phase induction motor supplied by a voltage source inverter, as shown in Figure 3. Some photographs of the elements contained in the system are shown in Figure 4. Also, the parameters of the motor are provided in Table 1. The digital signal processor was the TMS320F28335 unit, a medium-power system capable of holding both the outer and inner control loops. The incremental encoder was the GHM510296R quadrature unit, providing 2500 × 4 pulses per revolution (ppr).

### 4.3. Performance Indices

As stated in the Introduction Section, the ultimate objective of the proposed speed sensing method is the improvement of the speed control of the drive. Several figures of merit were considered for the assessment of such speed control. Please notice that other performance indicators could be used for particular applications. However, the set presented below is commonly considered [34]. This is based on the closed-loop response of the system to a step change in the reference.

- Mechanical speed overshoot (PO). In most cases, some overshoot is allowed as a trade-off with the speed of response (i.e., short transients).
- Rise time ($T_{r}$). It is defined as the time from the step change in reference to the first arrival at the new level. Short transients are characterized by low values of $T_{r}$ followed by well-damped behavior.
- Integral time absolute error (ITAE). This indicator takes into account how long the oscillations last after a step change in the reference. For a well-damped system, the ITAE should be low.

**Figure 5:**
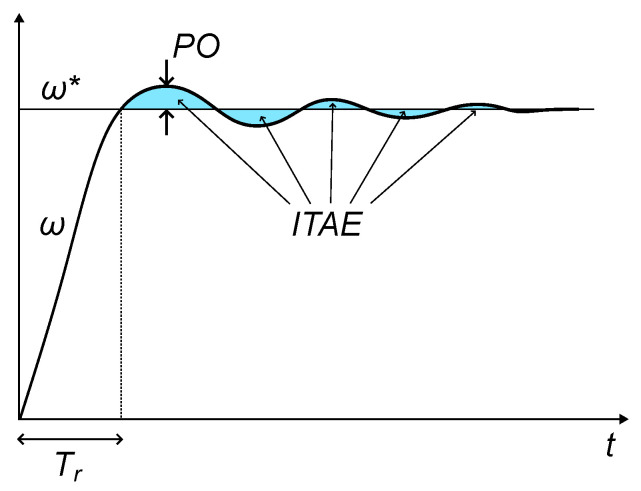
An illustration of the performance indices $\gamma_{i}$.

These indices are illustrated by Figure 5, where the trajectory of speed is presented after a step change in the reference. They can be defined by the following expressions.

(8) $$\begin{array}{ccc} PO & = & 100\cdot \frac{\max\omega (k)-\omega^{*}}{\Delta \omega^{*}} \end{array}$$

(9) $$\begin{array}{ccc} T_{r} & = & T_{s}\cdot \left(\underset{k\geq 0}{\mathrm{argmin}}\;\omega^{*}-\omega \left(k\right)\right) \end{array}$$

(10) $$\begin{array}{ccc} ITAE & = & \frac{1}{N}\sum_{k=1}^{N}|\omega^{*}-\omega \left(k\right)|\cdot k \end{array}$$

In the above expressions, $T_{s}$ is the sampling period of the DSP control routine, $\omega^{*}$ is the speed reference after the step change, and the discrete time at which the step takes place is set to be k=0.

### 4.4. Tests and Results

The experiments were carried out with the experimental setup presented earlier. The actual tests consisted of step changes provided to the reference speed. In this way, the performance indicators PO, $T_{r}$, and ITAE could be measured for each case. The standard speed sensing method was tested and compared to the proposal. The same PI tuning was used for the proposal and the standard method to obtain a clear comparison. Please note that the proposal could potentially use different tunings due to the reduced time lag in the loop. The two cases for the comparison were for two PI tunings with the parameters shown below. These were found by trial and error so that a typical overshoot and rising time were found.

- Case (a), where $k_{p}=0.92$ and $k_{i}=10^{-4}$. This tuning aims at a trade-off between overshoot and damped response.
- Case (b), where $k_{p}=1.20$ and $k_{i}=1.8\cdot 10^{-4}$. This tuning aims at a faster response, potentially incurring higher oscillations.

**Figure 6:**
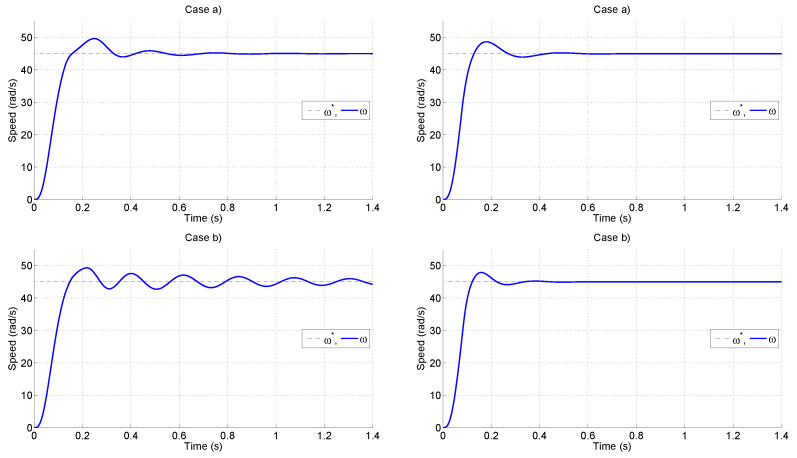
Trajectories of speed for the standard speed sensing method (**left**) and for the proposal (**right**) with two PI tunings: cases (a) (**top**) and (b) (**bottom**) described in the main text.

The resulting trajectories are shown in Figure 6. The reference speed suffered a step change at t=0. The actual speed for the standard speed sensing method is shown in the left column. The oscillatory behavior was a consequence of the time lag due to the standard sensing procedure. In particular, for case (b), the oscillations took longer to dissipate. Please note that for that same tuning (b), the proposal performed objectively better.

**Figure 7:**
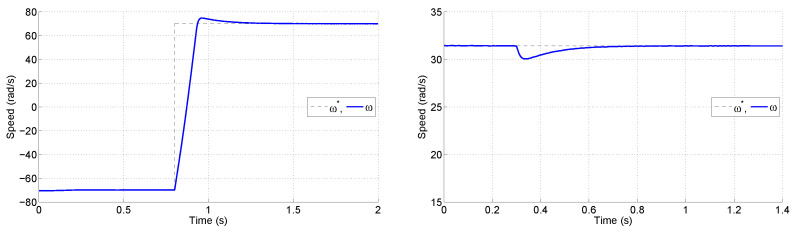
Trajectories of speed for the proposal during a reversal test (**left**) and a load-change test (**right**).

In addition to the graphical results of Figure 6, Table 2 presents the figures of merit for the tests. It can be seen that the proposal allowed for a reduction in all figures of merit. Please note that the trade-offs between figures of merit were still present; however, they were shifted to lower values thanks to the reduction in the time lag due to the sensing procedure. Also the results of a reversal test and a load-change are provided in Figure 7 for the proposal.

**Figure 8:**
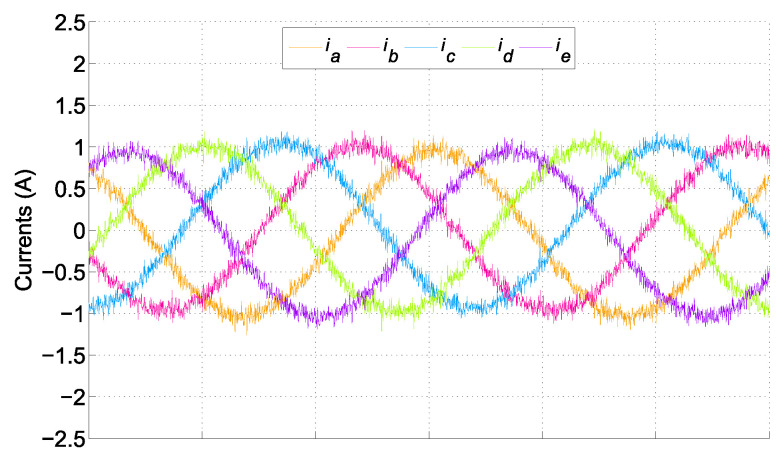
Stator current waveforms for the five-phase motor.

The stator phase currents in the sinusoidal steady state can be seen in Figure 8. Please note that electrical variables were handled by the inner loop, which featured a much shorter time scale. This means that within one electrical cycle, speed changed very little for both approaches.

## 5. Discussion

The results presented in this paper show an improvement in speed control. Compared to the pulse counting at fixed time intervals, the proposal was able to detect accelerations, thus reducing the inherent time lag. In the experiments, the actual values of the figures of merit depended on PI tuning. However, it was shown that the proposal achieved better results for each tuning.

It is important to realize that in most works dealing with PI tuning, the latency of speed sensing is not considered. As a result, tuning methods can produce inadequate results since the theory behind them assumes perfect knowledge of the mechanical speed. In this regard, the use of a better speed sensing method helps in achieving theoretical goals.

The improved results are a consequence of the proposal design in which both the variance of the sensed speed and the operating regime are considered. Notice that the pulse counting method must either resort to strong filtering (with increased latency) or to little filtering (with increased fluctuations in measurements).

Finally, the design of the proposal is compatible with previous DSP speed sensing methods that rely on interruption-based counting. This helps promoting its adoption in existing applications.

## 6. Conclusions

The addition of a transient detector to the basic speed estimation method has a positive effect on drive performance. This improvement is found without abandoning the commonly found paradigm of interruption-based pulse counting.

Future research work can be used to determine improvements derived for other drive control structures and to determine the best filtering strategy to work with the transient detector.

## Figures and Tables

**Table 1:** Parameters of the experimental five-phase drive.

Parameter	Value	Units
Resistance stator side, $R_{s}$	12.85	Ω
Resistance rotor side, $R_{r}$	4.80	Ω
Mutual inductance, $L_{M}$	681.7	mH
Number of pairs of poles, *P*	3	-
Encoder precision, *Q*	2500	ppr
Control sampling period, $T_{s}$	50	μs

**Table 2:** Comparison of figures of merit.

	Standard			Proposal		
Tuning	PO	$T_{r}$	ITAE	PO	$T_{r}$	ITAE
(a)	10.39	0.1569	50.6	8.13	0.1287	27.9
(b)	9.54	0.1475	328.4	6.40	0.1215	17.2

## Data Availability

The raw data supporting the conclusions of this article will be made available by the authors on reasonable request.

## Abbreviations

The following abbreviations are used in this manuscript:

ASF	Average switching frequency
DSP	Digital signal processor
FSMPC	Finite-State Model-Predictive Control
IFOC	Indirect Field-Oriented Control
IM	Induction machine
ITAE	Integral of time-weighted absolute error
MPC	Model-Predictive Control
PI	Proportional integral
PO	Percentage overshoot
PID	Proportional integral derivative
PWM	Pulse width modulation
VSI	Voltage source inverter
